# Bioprocessing of human platelet concentrates to generate lysates and extracellular vesicles for therapeutic applications^✰^

**DOI:** 10.1016/j.mex.2024.102822

**Published:** 2024-07-09

**Authors:** Wei-Ting Yeh, Ezrin Yi-Ling Yu, Ya-Hsuan Lu, Dora Livkisa, Thierry Burnouf, David J. Lundy

**Affiliations:** a School of Biomedical Engineering, Taipei Medical University, 301 Yuantong Road, Taipei 235603, Taiwan; b International PhD Program in Biomedical Engineering, Taipei Medical University, 301 Yuantong Road, Taipei 235603, Taiwan; c Graduate Institute of Biomedical Materials & Tissue Engineering, Taipei Medical University, 301 Yuantong Road, Taipei 235603, Taiwan

**Keywords:** Biomaterial, Exosome, Size exclusion chromatography, Blood-based therapeutic, Bioprocessing, Bioprocessing of human platelet concentrates to generate lysates and isolation of platelet-derived extracellular vesicles

## Abstract

This work describes protocols for preparing specific forms of human platelet lysates from pooled platelet concentrates (PCs) and the isolation of platelet-derived extracellular vesicles (p-EVs). Clinical-grade PCs can be sourced from blood establishments immediately following expiration for transfusion use. Here, we describe methods to process PCs into specific lysates from which p-EVs can be isolated. Each lysate type is prepared using platelet activation and processing methods which produce distinct products that may be useful in different applications. For example, serum-converted platelet lysate (SCPL)-EVs were recently shown to have powerful therapeutic properties following myocardial infarction in mice. EVs can be isolated from all products using size exclusion chromatography, producing pure and consistent p-EVs from multiple batches. Together, these methods allow isolation of p-EVs with excellent potential for clinical and preclinical applications.

• Platelet concentrates (PCs) obtained from local blood establishments are reliable and sustainable sources to generate biomaterials.
• We outline five distinct methods of platelet lysate generation and one method for extracellular vesicle isolation.
• Each platelet lysate form has different biological properties which may be suitable for certain applications.

## Graphical abstract

**Figure:**
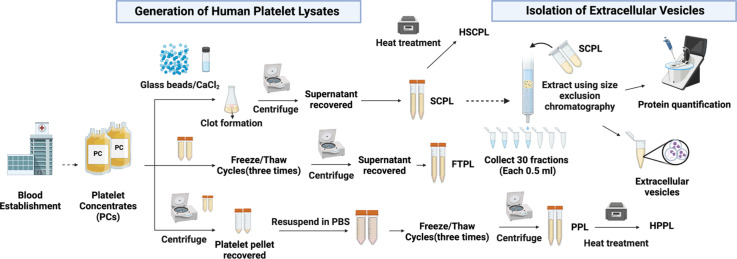

Specifications table

**Table:** 

Subject area:	Biomedical Sciences
More specific subject area:	Extracellular Vesicle Research and Platelet Lysate Bioprocessing
Name of your method:	Bioprocessing of human platelet concentrates to generate lysates and isolation of platelet-derived extracellular vesicles
Name and reference of original method:	Delila, L., et al., Extensive characterization of the composition and functional activities of five preparations of human platelet lysates for dedicated clinical uses. Platelets, 2021. 32(2): p. 259–272.
Resource availability:	Bioprocessing of human platelet concentrates to generate lysates: • Platelet concentrates sourced from local blood establishments • Activation reagents: 0.22 µm- filtered 0.9 M CaCl_2_ • 2 mm diameter sterile glass beads Isolation of platelet-derived extracellular vesicles: • Size exclusion chromatography setup • qEV original 35 nm Gen 2″ (Izon, ICO-35–100). • Storage buffer: 20 % ethanol • CIP buffer: 0.5 M NaOH

## Background

Allogeneic platelet concentrates (PCs), obtained from healthy donors, are an important therapeutic product for managing or preventing bleeding among patients who suffer from low platelet counts or dysfunctional platelets. PCs consist of concentrated platelets from several donors, and are typically suspended in plasma or a mixture of plasma with additive solution [1]. The presence of plasma in PCs provides additional proteins and clotting factors that support the coagulation function of transfused platelets. However, if using PC-derived materials for therapy of some types of acute injury, disrupting or stimulating endogenous coagulation would be undesirable, whereas for other indications, like skin wound healing, fibrino stimulation is therapeutically favorable. Therefore, this manuscript describes several methods for processing PCs into platelet lysates and isolating platelet-derived extracellular vesicles (P-EVs).

**Fig. 1:**
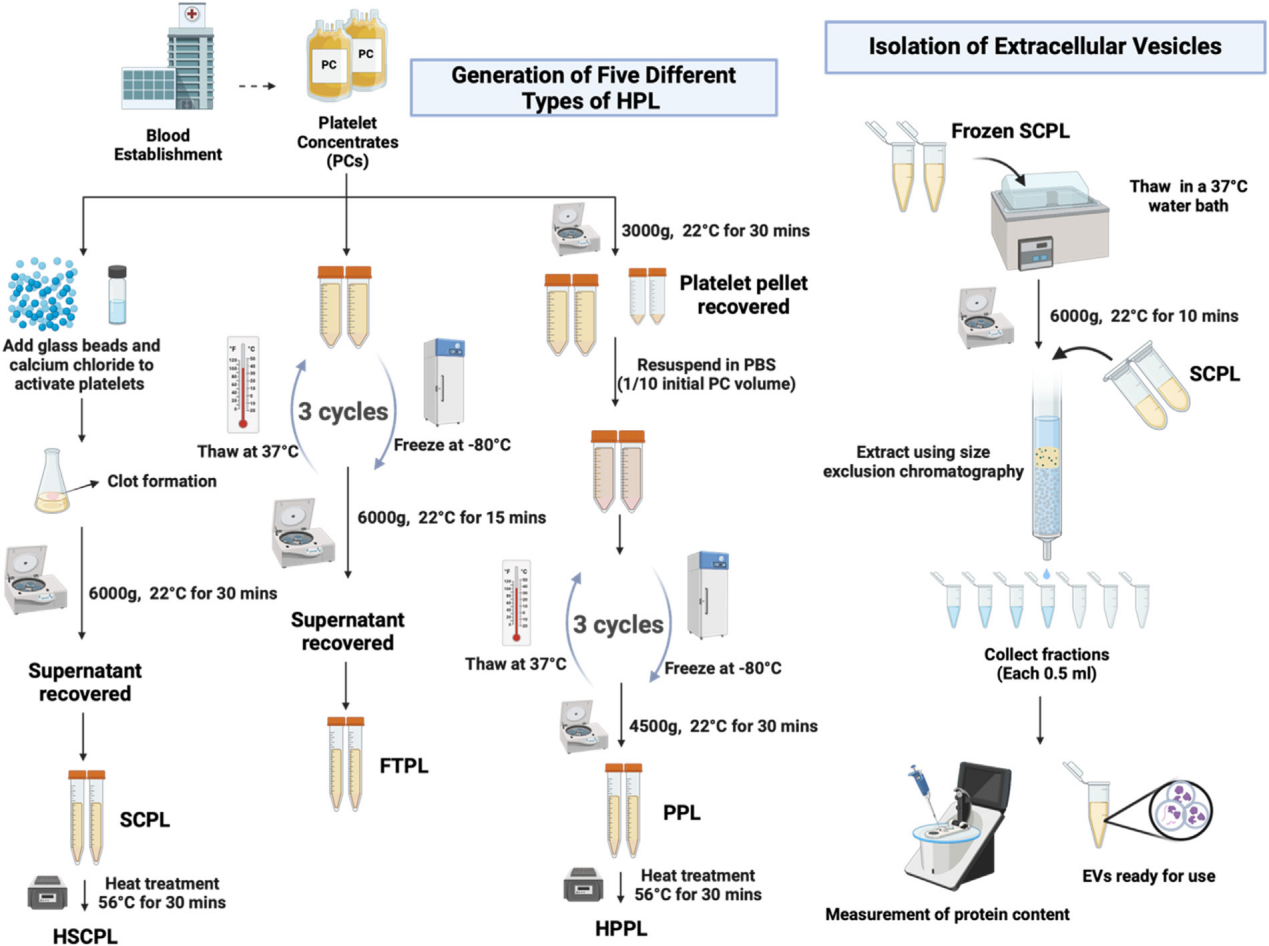
Overview of experimental procedures resulting in SCPL, FTPL and HPPL, and subsequent P-EV isolation. The schematic diagram illustrates the workflow of generating platelet lysates from platelet concentrates obtained from blood establishments. Two methods use platelet concentrates directly, activating and lysing them by calcium (SCPL) or freeze-thaw cycles (FTPL) respectively. A third process first concentrates the platelets into a pellet to reduce plasma components of the final product. Finally, isolation of platelet extracellular vesicles (p-EV) is shown. Created in Biorender.com.

Recognizing the therapeutic potential of platelet-based biomaterials, many researchers, including our group, have explored the use of platelet concentrates (PCs), platelet lysates, and more recently, platelet-derived microvesicles and P-EVs in various pre-clinical indications [[2], [3], [4], [5], [6]]. Platelets are known to release a distinctive assortment of extracellular vesicles (p-EVs) into the bloodstream, which are packed with diverse and influential biomolecular content crucial for intercellular communication [7]. Therefore, the activation and lysis of platelets are important steps to facilitate the release of these trophic factors and complement platelet function in coagulation or tissue repair [8]. In terms of therapeutic potential, several studies highlight the significant therapeutic possibilities of platelet-derived EVs. For example, our research group recently showed that EVs from serum-converted platelet lysates (SCPL-EVs) protected both rodent and human cardiomyocytes from hypoxia-induced damage and promoted angiogenesis in mouse myocardial infarction models with reperfusion [9]. Other research has demonstrated their efficacy in accelerating wound healing in diabetic rat models and repairing damage to the corneal endothelium and tendons [10,11]. Recently, a first-in-human trial using p-EVs in a skin wound healing context was published, demonstrating the possibility of clinical translation [12]. Methods used for platelet bioprocessing influence the cargo and function of the resulting lysates [6,13]. In this work, we describe a selection of five specific platelet lysate types used in our labs; freeze-thaw platelet lysate (FTPL), platelet pellet lysate (PPL), heated platelet pellet lysate (HPPL), serum converted platelet lysate (SCPL), and heated serum converted platelet lysate (HSCPL). We also describe the isolation of EVs using size exclusion chromatography, using SCPL-EVs as an example. These methods have been selected to provide researchers with a diverse selection of options, informed by our experience in the area. These include published proteomic and miRNA datasets from resulting products, the ability of each method to preserve the bioactivity of growth factors, and our in-house testing which finds that lysate preparation function varies between applications. Alongside the methods, we give suggestions about potential uses of each product. Lastly, in making the selection, we considered the yield of EVs, the degree of reproducibility, and the feasibility for translating to clinical applications. A schematic overview of the protocols is shown in Fig. 1.

## Method details

### Considerations for platelet concentrate collection and handling

Our protocol for the bioprocessing of human platelet concentrates into lysates and the isolation of platelet-derived extracellular vesicles (p-EVs) consolidates established methodologies into a comprehensive, standardized framework. It is known that different bioprocessing methods result in platelet lysates with different compositions. In this manuscript, we provide researchers with a simple, standardized approach for generating five distinct types of platelet lysates, each tailored for specific therapeutic applications. In particular, the methods have been developed to enhance reproducibility and facilitate scalability for preclinical or future clinical applications. We provide guidance for rapid, reproducible isolation of high-purity extracellular vesicles from platelet lysates. The end products have been validated in terms of proteomic content, p-EV miRNA cargo, and p-EV function using *in vitro* and *in vivo* models. Additionally, we provide advice for further characterization and downstream experimental use of p-EVs.

The starting materials for this protocol are pooled platelet concentrates (PCs), obtained from a local blood establishment. For our pre-clinical research, these PCs are obtained immediately after passing the expiration date for transfusion use; thus, they are waste products which would be otherwise discarded. As such, good communication with the blood establishment is very helpful so that they may give researchers advance notice of upcoming expiring PCs. Users may also wish to ask the blood establishment about their specific methods for platelet isolation, PC generation and donation pooling, since procedures may vary.

Although using otherwise waste materials does not pose significant ethical issues, researchers should still obtain institutional review board (IRB) approvals for the use of human-derived tissues. Ideally, the blood establishment handles donor recruitment, screening, informed consent that the donation may be used for research if not transfused, compensation or remuneration (if applicable), sample collection for safety testing and PC generation. Researchers then obtain anonymized clinical-grade products soon after expiry for transfusion. In our labs, PCs are obtained from our local blood establishment immediately following expiry. Each PC is a bag of approximately 200 ml containing platelets from 5 to 7 separate donors suspended in plasma and anticoagulated in a citrate solution. If desired, the PCs can be pooled again to increase the number of donations contributing to the pool. In the related research article for this method, three PCs were pooled to create a larger batch of 15–18 donations from different donors. This has been previously shown as sufficient to create a standardized product in terms of protein content and ability to support MSC proliferation [14].

### Converting platelet concentrate into serum converted platelet lysate (SCPL)

This method uses calcium to activate the blood coagulation, induce fibrin formation, degranulate the platelets and induce EV release [15,16]. SCPL contains chemokine (C-X-C motif) ligand-16 (CXCL-16), prolactin, and tissue inhibitors of metalloproteinase-4 (TIMP-4), and vascular endothelial growth factor (VEGF). Suitable applications of SCPL include the generation of platelet-based gels for wound healing and bone growth [17,18]. Our previous research found several differences between the whole SCPL and SCPL-EVs. Both SCPL and SCPL-EVs contain EGF, IGF-1 and angiopoietin-1. However, SCPL is relatively anti-angiogenic, inhibited cardiac endothelial cell tube formation, and was found to contain more anti-angiogenic plasminogen, endostatin, chemokine (C-C motif) ligand 5 CCL-5 and CXCL-4 (also termed platelet factor 4) than SCPL-EVs. SCPL-EVs were more pro-angiogenic, likely due to removal of anti-angiogenic factors. Both SCPL and SCPL-EVs polarized naïve macrophages towards an “M2” anti-inflammatory phenotype [9]. Culturing MSCs in calcium-activated platelet lysates enhances their immunomodulatory effects [16].

To generate SCPL:

1. Place the PC bag on a waver shaker (60 rpm) for 30 min at room temperature.
2. Add between 10 ml and 30 ml of PC into a 50 ml tube.
3. For each 10 ml of PC, add 0.3 ml of filtered (0.22 µm) 0.9 M CaCl_2_ to reach a final concentration of 23 mM.
  -Calcium activates platelets and induces release of EVs.
4. Simultaneously, add 5 g of 2 mm diameter sterile glass beads per 10 ml PC.
  -Glass beads stimulate the coagulation process and facilitate in fibrin clot removal
5. Seal the tube and slowly rotate the mixture at room temperature until white-colored particulate suspensions become visible. This is usually within 1 hour.
  -The precipitates are fibrin clots
6. Following clot formation, rotate the mixture for additional 30 min at room temperature
  -This consolidates the adhesion of the fibrin clot to the glass beads and achieve complete release of the platelet granule factors.
7. Centrifuge the 50 ml tube at 6000x g for 30 min at room temperature and collect the supernatant into a new tube.
8. Centrifuge again at 6000x g for 30 min.
9. Collect the supernatant (SCPL).
10. Aliquot and store the SCPL at −80 °C until use.

SCPL can be applied directly to cells and we suggest a starting point of 1% (v/v) SCPL/culture medium. SCPL contains residual calcium, thus a negative control group including the corresponding concentration of CaCl2 should be included.

### Converting SCPL into heated serum converted platelet lysate (HSCPL)

Considering that the calcium treatment to prepare SCPL leads to an activation of the blood coagulation and thus the generation pro-coagulant thrombin and proteolytic enzymes, as well as an activation of the complement system, it can be beneficial to perform a heat-treatment to inactivate potentially detrimental factors and improve treatment safety in some indications.

1. Place the SCPL tube into a water bath at 56 ± 1 °C for 30 min.
2. Centrifuge the heat-treated SCPL at 6000 x g for 30 min at 22 ± 2 °C and collect the supernatant into a new tube.
3. Centrifuge again at 6000x g for 30 min at 22 ± 2 °C.
4. Collect the supernatant (HSCPL), aliquot, and store at −80 °C until further use.

### Converting platelet concentrate into freeze-thaw platelet lysate (FTPL)

This method uses cycles of freezing and thawing to lyse the platelets and release the content. This avoids use of calcium (such as in SCPL), which may be desirable, since calcium can affect downstream assays and biological function of the platelet lysate. However, without removal of fibrinogen, FTPL can induce gelation when added to culture medium. This can be prevented by addition of heparin; however, heparin also has biological activity, such as binding to some platelet growth factors, which may impact downstream assays. FTPL contains, as expected, higher total protein and higher amounts of IGF-1 than PPL and HPPL. FTPL has previously been shown as suitable for joint healing [15].

1. Place the PC bag on a waver shaker (speed at 60 rpm) for 30 min at room temperature
2. Add between 10 ml and 30 ml of PC into a 50 ml tube
3. Place the tube into a -80 ± 1 °C freezer until completely frozen. This requires typically at least 80 min. If the solution is not completely frozen upon checking, return it to the freezer.
4. Thaw the mixture in a water bath set to 37 ± 1 °C for 30 min. Gently invert the tube five times.
5. Repeat step 3 and step 4 for an additional 2 times.
  -Three freeze-thaw cycles ensure complete lysis of the platelets and release of their contents
6. Centrifuge at 6000 x g for 30 min at 22 ± 2 °C, repeat if the supernatant isn't clear
  -This is done to remove cell debris.
7. Collect the supernatant into a new tube
8. Aliquot the supernatant (FTPL) and store at −80 °C until further use.

### Converting platelet concentrate into platelet pellet lysate (PPL)

As mentioned earlier, PCs include platelets suspended in plasma with or without platelet additive solution. This method uses centrifugation to concentrate platelets and deplete plasma proteins. The platelets are then lysed using freeze-thaw cycles. PPL is enriched in platelet-derived growth factors, and the protein composition differs greatly from other lysates due to the removal of the protein-rich plasma supernatant. Removing plasma contributes to depleting the lysate from proteins such as fibrinogen, activated coagulation factors, or proteolytic enzymes, that can be detrimental for brain administration. Recent studies show that overexpression of C3 and C4 triggers exacerbation of inflammation in Alzheimer's disease and other neurological disorders, ultimately resulting in neuronal loss. Due to its low C3 and C4 content, PPL could be a more suitable option for brain administration [15].

1. Place the PC bag on a waver shaker (speed at 60 rpm) for 30 min at room temperature
2. Add between 10 ml and 30 ml of PC into a 50 ml tube.
3. Centrifuge at 3000 x g for 30 mins at 22 ± 2 °C.
4. Carefully remove the plasma supernatant from the platelet pellet with an automatic pipette.
5. Wash the surface of the pellet by gently adding, then removing, 1 ml of sterile PBS, centrifuge again
  -we recommend slowly pipetting the PBS onto the inner wall of the tube, not directly onto the pellet
  -if the pellet is accidentally disturbed, repeat the centrifugation step
6. Add PBS at 10 % (v/v) of the initial PC volume.
7. Gently pipette up and down to resuspend the pellet.
8. Subject to three freeze-thaw cycles (−80 °C until frozen / 37 ± 1 °C for 30 min).
9. Centrifuge at 4500 x g for 30 min at 22 ± 2 °C to remove cell debris.
10. Aliquot the supernatant into a new tube and repeat the centrifugation to remove platelet debris.
11. Aliquot the PPL and store at −80 °C until further use.

### Converting platelet concentrate into heated platelet pellet lysate (HPPL)

Heat treatment removes fibrinogen and inactivates immunological components released from the platelets. We have found that this treatment unexpectedly enhances neuroprotective properties as well as reduces toxicity to neuronal cells, making it suitable for treating neurological disorders [19]. There was no IGF-1, endostatin, or endoglin detected in HPPL. However, HPPL contains a higher relative level of TGF-β compared to other four kinds of PLs, even PPL. Moreover, it is one of only two PLs that found B-cell activating factor (BAFF) and myeloperoxidase [15].

1. Starting with PPL, heat treat PPL by placing in a water bath at 56 ± 1 °C for 30 min.
2. Immediately cool for at least 5 min on ice.
  -to break down the pellets
3. Centrifuge at 10^4^ × *g* for 15 min at 4 ± 2 °C.
  -To eliminate any precipitation.
4. Store HPPL at −80 °C until further use [20].

### Isolation of extracellular vesicles from serum converted platelet lysate

In the following section, SCPL will be used as an illustrative example to demonstrate the isolation of extracellular vesicles (SCPL-EVs) based on the reference publication. While the protein yield, released factors and EV cargo will vary between each platelet preparation, the principles of size exclusion chromatography are consistent for all products. Each form of platelet lysate contains multiple components including EVs (80–200 nm vesicles) and varying proportions of proteins such as albumin, immunoglobulins, platelet proteins and growth factors. Size exclusion chromatography can effectively separate EVs from other proteins since they are much larger and will elute from the column earlier, at its exclusion volume [21]. In the following protocol we use a commercial size exclusion chromatography system, but alternatives can be established using agarose, Sephadex or other products [22]. Each fraction can be measured using a combination of analytical techniques to determine which contain the highest concentration of EVs and which contain free proteins.

### Size exclusion chromatography

1. Thaw the frozen SCPL aliquot at 37 °C until it is completely thawed with no visible ice crystals.
2. Centrifuge the sample at 6000 g for 10 mins at 4 °C to remove any insoluble that sediment or appear as a white layer on the top of the supernatant.
3. Prepare sufficient storage buffer and CIP (cleaning in place) buffer for the planned experiments, as shown in the table below. Both buffers should be sterilized by passing through a 0.2 µm syringe filter. Unused buffers can be stored at room temperature for future use. If the CIP buffer develops precipitates, heat the solution and mix to dissolve the precipitate, then filter again before use.

**Table:** 

Buffer	Ingredient	Solvent
Storage buffer	20 % ethanol	0.1 um filtered DPBS
CIP buffer	0.5 M NaOH	0.1 um filtered DPBS

4. For the reference publication we used “qEV original 35 nm Gen 2″ (Izon, ICO-35–100). Remove the top cap and attach the column in an upright position to a stand with a collection tube underneath.
  -Note: The following instructions are based on a column optimized for 500 µl samples. Larger or smaller capacity columns require different sample and elution volumes.
5. Remove the bottom cap. The storage buffer inside the column will begin to flow out.
  -do not allow the liquid level to fall below the resin.
6. Before the storage buffer reaches the resin surface, add 20 ml of 0.1 µm filtered DPBS to the column.
7. Prepare four tubes and begin collecting the flow-through as soon as it starts to drip from the column. Collect 5 ml per tube and continue until all four tubes are filled.
8. Measure the pH of the contents in the third and fourth tubes. If both samples have a pH of 7.2 +/- 0.1, identical to that of the 0.1 µm filtered DPBS, this indicates that the column has been successfully equilibrated. If the pH is not within this range, continue to equilibrate the column with the filtered DPBS until the pH of flowthrough of the column reaches the intended pH range.
9. Add 0.5 ml of the platelet lysate (SCPL, FTPL *etc.*) sample to the column.
10. Once the sample penetrates into the resin, start slowly adding filtered DPBS.
11. Arrange 1.5 ml Eppendorf tubes in a suitable rack before starting the elution. 20 to 40 are recommended, depending on the number of fractions to be captured.
12. Begin collecting the eluate as soon as it starts to drip from the column. Sequentially fill each Eppendorf tube with 0.5 ml of the eluate, continuing until all tubes are filled.
  -In the example shown below, 20 fractions were collected, since these contain the components most relevant to our experiments. Additional fractions can be collected as required.
13. Inject 20 ml of 0.5 M CIP buffer and allow it to flow through to wash the column
14. Inject 20 ml of 0.1 µm-filtered DPBS and allow it to flow through to equilibrate the pH.
15. When the experiment is complete, inject 20 ml of storage buffer and put the column in a 4 °C refrigerator.

### Analysis of size exclusion chromatography fractions

There are many possible analyses which can be performed on the fractions. Here, we provide guidance.

1. Determine the protein concentration of each fraction. This can be done using protein assays (BCA, Bradford etc.) or approximated using spectrometry at 280 nm. In our experience, Nanodrop or similar devices are suitable for this. A typical result is shown in Fig. 2. The first four fractions are DPBS flowthrough. Subsequent fractions contain a low amount of protein, and the majority of proteins are eluted between fractions 12 and 18, with the peak between fractions 13–16. After fraction 20, very little protein is eluted from the column.

**Fig. 2:**
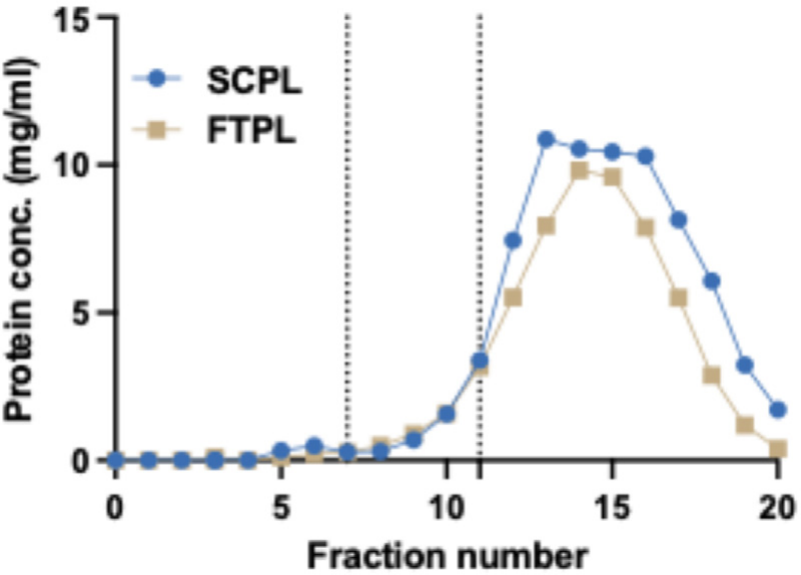
Protein concentration of fractions from SCPL and FTPL. The plot shows protein concentrations (Y axis) approximated by spectrophotometry at 280 nm for SCPL (blue) and FTPL (gold) following size exclusion chromatography. 20 fractions are shown (X axis). Based on the principles of size exclusion chromatography, the first four fractions are mostly DPBS flowthrough, thus they have a low/undetectable protein concentration. Low levels of protein appear from fraction 5 onwards and begins to rise after fraction 10. A large amount of protein is then eluted from the column, peaking between fractions 13–16. These are free proteins. The dotted lines on the graph illustrate the selection of SCPL-EV and FTPL-EV fractions for these samples. Different fractions can be selected by end users, balancing purity against yield, depending on their intended purpose.
2. Determine the particle size distribution of every fraction, or selected fractions. Nanoparticle tracking analysis (NTA) or dynamic light scattering (DLS) are both suitable methods. NTA gives additional information about the particle concentration, but is more time-consuming than DLS. Fractions 7–11 contain a high concentration of particles in the 80 to 120 nm size range. Therefore, these fractions are likely to contain P-EVs. For most studies, we opt to pool these fractions. However, each fraction could be explored separately if desired. Later fractions (> 10) have reduced particle sizes of < 20 nm and significantly higher protein concentrations, indicating that they contain mostly free proteins.
3. Electron microscopy, particularly cryoEM can be used to visually inspect fractions. These can support particle size measurements from NTA or DLS. Additionally, they allow for gold standard confirmation of EV presence by visualizing the lipid bilayer membrane, distinguishing EVs from other particles. Example images of SCPL fractions are shown in Fig. 3.

**Fig. 3:**
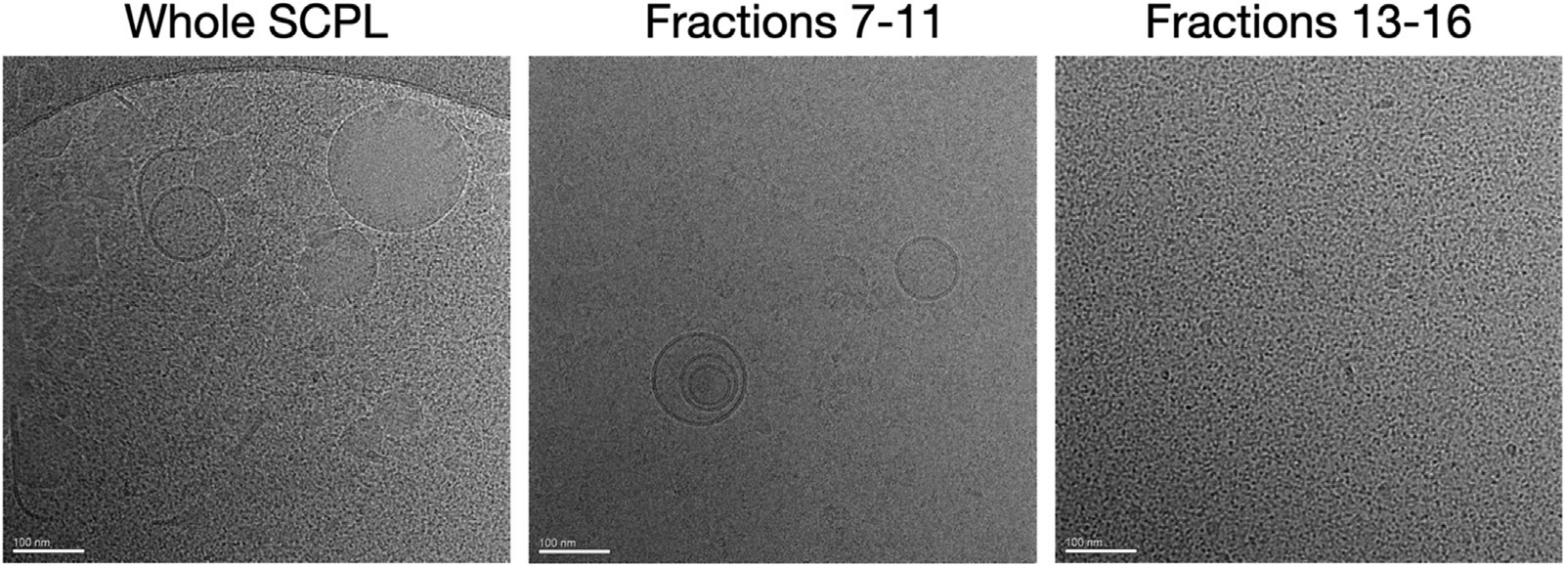
CryoEM of platelet lysate and size exclusion chromatography fractions. Under cryoEM, differences between fractions are easily seen. The whole SCPL (i.e. before size exclusion chromatography) contains a mixture of EVs and other nanoscale particles. These are likely lipoproteins, and can be distinguished from EVs by the lack of lipid bilayer. In addition, note the granular appearance of the background, which is likely free proteins. Fractions 7–11 contain abundant 80–120 nm vesicles. The lipid bilayer membrane is clearly visible, distinguishing them from other similarly-sized particles. EVs can be either unilamellar or multilamellar, as shown in the image above. Note the relatively clean background of the image compared to whole SCPL. Fractions 13–16 do not contain vesicles or other larger particles. These later fractions contain abundant granular particles which are likely free proteins.
4. ELISA can be used to measure concentrations of common non-EV components such as apolipoproteins, albumin and immunoglobulins in the EV and protein fractions. However, it should be noted that some of these proteins may be part of the EV surface corona, and thus form part of their biological function. Thus, it may not be accurate to consider these as simply contaminants.
5. Successful EV isolation can be validated by measuring known EV marker proteins such as CD9, CD81, ALIX, TSG101, syntenin-1 and others. These proteins are associated with EV membranes and their biogenesis.
6. Platelet membrane proteins such as CD41 (GPIIb), CD42b (GPIb), CD61 (GPIIIa), or CD62P (P-selectin) can be assessed. These proteins allow platelets to interact with other cell types and participate in their biological functions of thrombosis and immune modulation. CD42 allows for platelet binding to endothelial cells, by targeting von Willebrand factor. CD62P allows binding with leukocytes through attachment to PSGL-1, assisting in the localization of platelets to injury sites, and their subsequent retention. CD41/CD61 are platelet-specific markers and can be used to demonstrate that the EVs originate from platelets. [1] Thus, these markers may be particularly relevant for researchers interested in p-EV targeting or studies utilizing p-EVs for drug delivery.
7. The particle to protein ratio can be a useful proxy for EV purity to compare batches. Since EVs make up only a small fraction of plasma and platelet protein, a higher particle:protein ratio indicates a higher purity of EV isolation [23]. Where possible, this should be reported.
8. Proteomic studies can be useful to determine the most abundant proteins in particular selection of fractions.
9. EV miRNAs appear to be important for their bioactivity. In our experience with SCPL-EV miRNA measurements, we found that different miRNA isolation methods produce very variable yields and purity. We obtained the most reproducible results using qEV miRNA isolation kits [Izon, RXT01] and quantification using the PCR-based Qiagen LNA system. We strongly recommend using spike-in synthetic miRNAs to validate successful miRNA isolation, reverse transcription and linear amplification.
10. Functional analyses can be performed on cultured cells. This may include endothelial cell migration or tube formation assays, cell protection (hypoxia, drug toxicity, or other injury stimulus) assays, immune cell activation. For platelet lysates, we recommend a starting concentration of 1 % v/v to screen for activity. Dosing based on protein concentration should be determined. For isolated EVs, dosing can be based on protein concentration or particle count. Note: P-EVs may contain enzymes. Therefore, for viability assays (MTT, WST, CCK-8) or cell injury assays (LDH) running a blank sample of culture medium and P-EVs, without cells, is essential. This value can be subtracted from results obtained using cells.
11. For some studies, the fractions from size exclusion chromatography may be too dilute, depending on the doses needed for downstream applications. EVs can be concentrated using centrifugation columns, choosing an appropriate membrane cutoff size. However, this will result in loss of some EVs into the filter. Ultracentrifugation may also be used to concentrate EVs into a pellet.

## Limitations

None.

## Ethics statements

For the use of human materials (platelet concentrates) Institutional Review Board approval was obtained from 10.13039/501100004700Taipei Medical University (Protocol: TMU-JIRB N201802052, Principal investigator: Thierry Burnouf).

## CRediT authorship contribution statement

**Wei-Ting Yeh:** Investigation, Formal analysis, Visualization, Writing – original draft. **Ezrin Yi-Ling Yu:** Investigation, Writing – original draft. **Ya-Hsuan Lu:** Investigation, Writing – original draft. **Dora Livkisa:** Investigation, Writing – original draft, Writing – review & editing. **Thierry Burnouf:** Conceptualization, Resources, Funding acquisition, Supervision, Writing – review & editing. **David J. Lundy:** Conceptualization, Resources, Funding acquisition, Supervision, Writing – original draft, Writing – review & editing.

## Declaration of competing interest

The authors declare the following financial interests/personal relationships which may be considered as potential competing interests:

Thierry Burnouf is a co-inventor of patents relating to use of platelet lysates for therapeutic purposes, owned by Taipei Medical University, Taiwan, and the University of Lille, France. Thierry Burnouf has financial interest and is co-founder of Invenis Therapeutics which develops platelet lysate products for neurological disorders. The company did not guide or fund the study. The other authors have no conflicts of interest to declare.

## Data Availability

No data was used for the research described in the article.

## Appendix Supplementary materials

Image, application 1
