# Supplementary material for: Bioprocessing of human platelet concentrates to generate lysates and extracellular vesicles for therapeutic applications
Source: MethodsX. 2024 Jul 9;13:102822. doi: 10.1016/j.mex.2024.102822 (PMC11299553; doi:10.1016/j.mex.2024.102822)
Supplement: Supplementary file 1 [file mmc1.docx]

**Supplementary material**

**Detailed Introduction**

Platelets, or thrombocytes, are nucleus-free, blood cells essential for stopping bleeding and participating in wound healing. Upon activation they release growth factors such as platelet-derived growth factor (PDGF), transforming growth factor beta (TGF-β), and vascular endothelial growth factor (VEGF), as well as other trophic factors including anti-inflammatory and antioxidant biomolecules. Following injury, these factors can provide acute protection of parenchymal cells, modulate the immune response, promote the formation of new blood vessels and contribute to wound healing and tissue repair[1, 2]. Platelets are readily harvested from whole blood, making them a convenient and valuable resource for medical applications.

Allogeneic platelet concentrates (PCs), obtained from healthy donors, are an important therapeutic product for managing or preventing bleeding among patients who suffer from low platelet counts or dysfunctional platelets. PCs consist of concentrated platelets from several donors, and are typically suspended in plasma or a mixture of plasma with additive solution[2]. The presence of plasma in PCs provides additional proteins and clotting factors that support the coagulation function of transfused platelets. However, if using PC-derived materials for therapy of some types of acute injury, disrupting or stimulating endogenous coagulation would be undesirable, whereas for other indications, like skin wound healing, fibrino stimulation is therapeutically favorable. Therefore, this manuscript describes several methods for processing PCs into platelet lysates and isolating platelet-derived extracellular vesicles (P-EVs). The plasma and platelet components of PCs can be processed together, or separated upstream before processing, resulting in lysates with distinct protein compositions or diverse P-EV populations which may be dedicated for different therapeutic purposes.

Recognizing the therapeutic potential of platelet-based biomaterials, many researchers, including our group, have explored the use of platelet concentrates (PCs), platelet lysates, and more recently, platelet-derived microvesicles and P-EVs in various pre-clinical indications[3-7]. In addition to the biological rationale, there are several practical advantages in using clinical grade PCs as a biomedical source material to develop therapies; they are produced by regulated blood establishments following stringent testing protocols. Thus, they are they are sterile, of consistent concentration and maximum level of residual red blood cells and white blood cells. In addition, pathogen-reduction procedures have been licensed and licensed in many countries. By contrast, most research using cell culture-derived EVs is performed using non GMP-grade reagents. Other advantages of P-EVs include reduced preparation time (and labor intensity) and high yields. For example, generating platelet lysates from PCs and separating P-EVs, described in this manuscript, can be completed within a few hours, whereas isolating cell-derived EVs requires several days of culture and preparing large volumes of conditioned serum-free culture medium[8]. Another advantage is that multiple donors can be easily combined. It is known that EV cargo and function varies between different source materials, between individual donors, and due to cell culture conditions[9]. Thus, it is challenging to standardize cell culture-derived EVs. However, PCs for adult use from most blood establishments contain platelets from 5-7 separate donors, and multiple PCs can be easily combined (or “pooled”), thus reducing the effects of individual donor variation on the final product. This is far less labor-intensive than pooling multiple cell culture-derived EVs. Lastly, platelets are known to release EVs during the period of storage prior to transfusion; thus we could consider that, de facto, unpurified p-EVs have already been given to millions of human patients worldwide.

Platelets are known to release into the bloodstream a distinctive assortment of extracellular vesicles (p-EVs), which are packed with diverse and influential biomolecular content crucial for intercellular communication[10]. Therefore, the activation and lysis of platelets are important steps to facilitate the release of these trophic factors and complement platelet function in coagulation or tissue repair[11]. The activation of platelets to produce lysates ex vivo can be achieved through multiple pathways, including freeze-thaw cycles, sonication, chemical or physical stimuli; with each method influencing the composition and yield of the lysate. Following platelet activation, P-EVs can be isolated from platelet lysates using techniques such as differential ultracentrifugation, size-exclusion chromatography, and filtration, each again offering different yield, throughput, purity, and scalability[8].

In terms of therapeutic potential, recent studies highlight the significant therapeutic possibilities of platelet-derived EVs. For example, our research group recently showed that EVs from serum-converted platelet lysates (SCPL-EVs) protected both rodent and human cardiomyocytes from hypoxia-induced damage and promoted angiogenesis in mouse myocardial infarction models with reperfusion[12]. Other research has demonstrated their efficacy in accelerating wound healing in diabetic rat models and repairing damage to the corneal endothelium and tendons[13, 14]. Recently, a first-in-human trial using p-EVs in a skin wound healing context was published, demonstrating the possibility of clinical translation[15].

P-EVs appear to act on multiple axis of the response to injury including immune modulation[16], energetics[6], parenchymal cell protection and angiogenesis[12]. P-EVs can also be exploited as drug delivery vehicles due to innate targeting from surface markers[5].

Methods used for platelet bioprocessing influence the cargo and function of the resulting lysates[7, 17]. In this work, we describe a selection of five specific platelet lysate types used in our labs; freeze-thaw platelet lysate (FTPL), platelet pellet lysate (PPL), heated platelet pellet lysate (HPPL), serum converted platelet lysate (SCPL), and heated serum converted platelet lysate (HSCPL). We also describe the isolation of EVs using size exclusion chromatography, using SCPL-EVs as an example. These methods have been selected to provide researchers with a diverse selection of options, informed by our experience in the area. These include published proteomic and miRNA datasets from resulting products, the ability of each method to preserve the bioactivity of growth factors, and our in-house testing which finds that lysate preparation function varies between applications. Alongside the methods, we give suggestions about potential uses of each product. Lastly, in making the selection, we considered the yield of EVs, the degree of reproducibility, and the feasibility for translating to clinical applications.

Reference:

1. Chong, D.L.W., et al., *Platelet-derived transforming growth factor-β1 promotes keratinocyte proliferation in cutaneous wound healing.* J Tissue Eng Regen Med, 2020. **14**(4): p. 645-649.

2. Burnouf, T., et al., *Expanding applications of allogeneic platelets, platelet lysates, and platelet extracellular vesicles in cell therapy, regenerative medicine, and targeted drug delivery.* Journal of Biomedical Science, 2023. **30**(1): p. 79.

3. Oeller, M., et al., *Human Platelet Lysate for Good Manufacturing Practice-Compliant Cell Production.* Int J Mol Sci, 2021. **22**(10).

4. Shih, D.T. and T. Burnouf, *Preparation, quality criteria, and properties of human blood platelet lysate supplements for ex vivo stem cell expansion.* N Biotechnol, 2015. **32**(1): p. 199-211.

5. Zhu, Y., et al., *Platelet-derived drug delivery systems: Pioneering treatment for cancer, cardiovascular diseases, infectious diseases, and beyond.* Biomaterials, 2024. **306**: p. 122478.

6. Pelletier, M., et al., *Platelet extracellular vesicles and their mitochondrial content improve the mitochondrial bioenergetics of cellular immune recipients.* Transfusion, 2023. **63**(10): p. 1983-1996.

7. Suades, R., et al., *Platelet-released extracellular vesicles: the effects of thrombin activation.* Cell Mol Life Sci, 2022. **79**(3): p. 190.

8. Lai, J.J., et al., *Exosome Processing and Characterization Approaches for Research and Technology Development.* Adv Sci (Weinh), 2022. **9**(15): p. e2103222.

9. Shekari, F., et al., *Cell culture-derived extracellular vesicles: Considerations for reporting cell culturing parameters.* Journal of Extracellular Biology, 2023. **2**(10): p. e115.

10. Puhm, F., E. Boilard, and K.R. Machlus, *Platelet Extracellular Vesicles: Beyond the Blood.* Arterioscler Thromb Vasc Biol, 2021. **41**(1): p. 87-96.

11. Goubran, H., et al., *Platelet and extracellular vesicles in COVID-19 infection and its vaccines.* Transfus Apher Sci, 2022. **61**(3): p. 103459.

12. Livkisa, D., et al., *Extracellular vesicles purified from serum-converted human platelet lysates offer strong protection after cardiac ischaemia/reperfusion injury.* Biomaterials, 2024. **306**: p. 122502.

13. Widyaningrum, R., et al., *In vitro evaluation of platelet extracellular vesicles (PEVs) for corneal endothelial regeneration.* Platelets, 2022. **33**(8): p. 1237-1250.

14. Graça, A.L., et al., *Therapeutic Effects of Platelet-Derived Extracellular Vesicles in a Bioengineered Tendon Disease Model.* Int J Mol Sci, 2022. **23**(6).

15. Johnson, J., et al., *First-in-human clinical trial of allogeneic, platelet-derived extracellular vesicles as a potential therapeutic for delayed wound healing.* J Extracell Vesicles, 2023. **12**(7): p. e12332.

16. Ilvonen, P., et al., *Distinct targeting and uptake of platelet and red blood cell-derived extracellular vesicles into immune cells.* Journal of Extracellular Biology, 2024. **3**(1): p. e130.

17. Nhi Thao Ngoc Le, C.-L.H., Liling Delila, Ouada Nebie, Hsin-Tung Chien, Yu-Wen Wu, Luc Buée, David Blum, and Thierry Burnouf, *Proteomics of human platelet lysates and insight from animal studies on platelet protein diffusion to hippocampus upon intranasal administration.* APL Bioengineering, 2024.
